# The kinase LRRK2 is required for the physiological function and expression of the glial glutamate transporter EAAT2 (SLC1A2)

**DOI:** 10.1111/jnc.16265

**Published:** 2024-12-10

**Authors:** Angela Di Iacovo, Chiara D'Agostino, Manan Bhatt, Tiziana Romanazzi, Stefano Giovannardi, Raffaella Cinquetti, Cristina Roseti, Elena Bossi

**Affiliations:** ^1^ Department of Biotechnology and Life Sciences, Laboratory of Cellular and Molecular Physiology University of Insubria Varese Italy; ^2^ Centre for Neuroscience University of Insubria Varese Italy; ^3^ PhD School of Experimental and Translational Medicine University of Insubria Varese Italy

**Keywords:** dopamine transporter, EAAT2 (SLC1A2), excitatory/inhibitory balance, LRRK2, SLC1, SLC6

## Abstract

Neurotransmitter transporters (NTTs) control synaptic responses by modulating the concentration of neurotransmitters at the synaptic cleft. Glutamate is the most abundant excitatory neurotransmitter in the brain and needs to be finely tuned in time and space to maintain a healthy brain and precise neurotransmission. The glutamate transporter EAAT2 (SLC1A2) is primarily responsible for glutamate clearance. EAAT2 impairment has been associated with Alzheimer's disease (AD), Huntington's disease (HD), amyotrophic lateral sclerosis (ALS), and Parkinson's disease (PD). Mutations in leucine‐rich repeat kinase 2 (LRRK2) contribute to both monogenic and sporadic forms of PD, of which the common substitution Gly2019Ser is associated with a significant deficit in EAAT2 expression. The role of pathological mutants of the LRRK2 is intensively studied and reviewed. Here we have focused the attention on the physiological role of LRRK2 on EAAT2, comparing the activity of NTTs with or without the LRRK2 kinase. By heterologous expression in *Xenopus laevis* oocytes and two‐electrode voltage clamp, the current amplitudes of the selected NTTs and kinetic parameters have been collected in the presence and absence of LRRK2. The results show that EAAT2 expression and function are impaired in the absence of the kinase and also under its pharmacological inhibition via MLi‐2 treatment. LRRK2 stabilizes EAAT2 expression increasing the amount of transporter at the plasma membrane. Interestingly, the LRRK2 action is EAAT2‐specific, as we observed no significant changes in the transport current amplitude and kinetic parameters obtained for the other excitatory and inhibitory NTTs studied. This study, for the first time, demonstrates the physiological importance of LRRK2 in EAAT2 function, highlighting the specificity of LRRK2‐mediated modulation of EAAT2 and suggesting a potential role for the kinase as a checkpoint for preserving neurons from excitotoxicity. In brain conditions associated with impaired glutamate clearance, targeting LRRK2 for EAAT2 regulation may offer novel therapeutic opportunities.

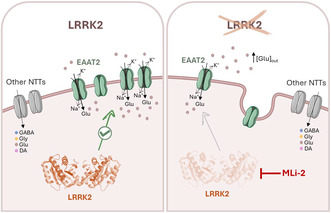

## Graphical abstract

The kinase LRRK2 promotes EAAT2 exposure on the plasma membrane, enhancing transporter availability and glutamate clearance activity. This effect is specific to EAAT2. In the absence or inhibition of LRRK2, low glutamate reuptake is observed.

**Abbreviations**
AD: Alzheimer's disease
ALS: amyotrophic lateral sclerosis
BGT‐1: betaine/GABA transporter
CNS: central nervous system
DA: dopamine
DAT: dopamine transporter
EAAT: excitatory amino acid transporter
E/I: excitatory/inhibitory
GABA: gamma‐aminobutyric acid
GAT: GABA transporter
Glu: glutamate
Gly: glycine
GlyT: glycine transporter
HD: Huntington's disease
LRRK2: leucine‐rich repeat kinase 2
NMDA: N‐methyl‐d‐aspartate
NT: neurotransmitter
NTT: neurotransmitter transporter
PD: Parkinson's disease
SLC: solute carrier
SOC: single oocyte chemiluminescence

## 1 INTRODUCTION

In neurons and glial cells, the neurotransmitter transporters (NTTs) control the neurotransmission through the reuptake process. The balance between glutamatergic and GABAergic networks in the central nervous system (CNS) ensures the proper and efficient processing of the information that is required for movement, cognition, and behavior (Sears & Hewett, 2021). The excitatory‐inhibitory (E/I) balance in CNS needs to be well‐maintained and tightly regulated. Any modification of this balance can lead to abnormal network activities, potentially resulting in various brain conditions (Ghatak et al., 2021; He & Cline, 2019; Pietropaolo & Provenzano, 2022). The increase in glutamate levels and the consequent over‐activation of glutamate receptors (GluRs) at the synaptic cleft, along with a reduction in GABA availability and decreased activation of GABA receptors, can contribute to neurodevelopmental and neurodegenerative disorders like Alzheimer's disease (AD; Takahashi, Kong, et al., 2015; Wood et al., 2022), Huntington's disease (HD; Bhatnagar et al., 2023), amyotrophic lateral sclerosis (ALS), and Parkinson's disease (PD; Iovino et al., 2022; Lewerenz & Maher, 2015). The excitatory amino acid transporters (EAATs) belonging to SLC family 1 (Solute Carrier) have been extensively studied investigating their role in the homeostasis of glutamate (Fahlke et al., 2016; Jensen et al., 2015; Kong et al., 2014). The SLC1 family is composed of seven proteins, including five EAAT proteins: EAAT1 and 2 are predominantly expressed in glial cells, EAAT3 is mostly in neurons, EAAT4 is the glutamate transporter of the Purkinje cells, and EAAT5 of the retinal cells (Takahashi, Foster, & Lin, 2015).

Recently, it has been reported that PD patients, carrying the most frequent mutation in LRRK2 with the substitution Gly‐Ser in 2019 position, show severe deficits in the expression of glutamate transporter EAAT2 (Iovino et al., 2022). The patients exhibit irregular communication between excitatory cortico‐striatal input and signal processing in the striatum. This disruption affects the normal flow of signals from the cerebral cortex to the striatum, leading to impaired motor control and coordination. In PD, the degeneration of dopaminergic neurons in the substantia nigra leads to E/I imbalance in this communication pathway (Helmich et al., 2010; Oran & Bar‐Gad, 2018; Skiteva et al., 2022).

Despite PD being the second most common neurodegenerative disorder worldwide, its physiopathology causing the clinical features remains unclear. The E/I imbalance, as a result of the increased synaptic excitation and the glutamate dyshomeostasis, is among the critical events responsible for some clinical symptoms reported in both genetic and sporadic PD (Iovino et al., 2020). The impairment of the membrane localization of EAAT2 caused by the mutated LRRK2 suggests a physiological role of the kinase in regulating the expression and activity of this membrane transporter (Iovino et al., 2022). Furthermore, patients with PD often display early neurochemical changes in the dopaminergic system before motor symptoms appear. The balance between dopamine (DA) release and reuptake determines the extracellular DA levels, a key factor in E/I balance at the basal ganglia (Barcomb & Ford, 2023). The loss of dopaminergic neurons is a hallmark of PD pathology. Notably, the PET imaging studies consistently demonstrate the increased DA turnover rates in asymptomatic LRRK2 G2019S heterozygotes, alongside reduced expression levels of dopamine transporter (DAT; Wile et al., 2016). Recently it was shown that the overexpression of LRRK2 G2019S in adult rats impaired the DAT‐mediated DA reuptake in an age‐dependent manner, suggesting that the impairment of the DAT activity is not strictly related to the mutated LRRK2 but is a consequence of progressive responses to genetic factors and age. These findings are also supported by behavioral changes happening only in elder transgenic rats, supporting the notion of gradual DAT dysfunction (Longo et al., 2017). Moreover, in vitro analysis did not reveal a direct interaction between mutant or wild‐type LRRK2 and DAT, indicating that the damage of DAT activity is likely an indirect effect of mutant LRRK2 (Zhou et al., 2011).

The primary inhibitory neurotransmitter, γ‐aminobutyric acid (GABA) contributes significantly to balancing the E/I ratio. Accordingly, the alterations in the activities of the GABA transporters (GATs) are reported in different neurodegenerative and neurodevelopmental disorders (Guerriero et al., 2015; Latka et al., 2020). In humans, GABA reuptake is regulated by the four identified GATs: GAT1‐3 and betaine/GABA transporter 1 (BGT‐1). The GATs are expressed both in neurons and glial cells, among them GAT1 is the most abundantly expressed in the CNS and extensively studied; whereas low levels of BGT‐1 are found in the brain, cerebral cortex, and cerebellum, it is primarily expressed in liver and kidney (Bhatt, Di Iacovo, et al., 2023; Bhatt, Gauthier‐Manuel, et al., 2023; Conti et al., 1999). Interestingly, the systematic supplementation of betaine, an endogenous osmolyte, is shown to have positive effects against several neurodegenerative and neuropsychiatric diseases, making BGT‐1 a potential target in maintaining E/I balance (Bhatt et al., 2024; Bhatt, Di Iacovo, et al., 2023).

Glycine transporters (GlyTs), including GlyT1 and GlyT2, regulate the balance between excitatory and inhibitory signals in the CNS by controlling glycine levels. Dysregulation, especially in GlyT1, impacts N‐methyl‐D‐aspartate (NMDA) receptor activity, leading to hyperexcitability (Harvey & Yee, 2013; Piniella & Zafra, 2023). While GlyT2 maintains glycine levels in the inhibitory neuron synapses (Brill et al., 2020). GlyTs act as key regulators in both excitatory and inhibitory neurons, emphasizing the significance of glycinergic activity in balancing neural signals (Eulenburg & Hulsmann, 2022).

The role and mechanisms of the action of LRRK2 are not fully understood. It is implicated in various cellular processes including the regulation of intracellular vesicle trafficking, autophagy, and lysosomal function. LRRK2 is a complex protein characterized by its dual enzymatic function, comprising serine–threonine kinase and GTPase activities. These enzymatic properties indicate LRRK2's involvement in intracellular signaling pathways. Moreover, the presence of distinct structural repeats, such as ankyrin, leucine‐rich, and WD40 domains located at the N‐ and C‐terminals, suggest that LRRK2 also serves as a scaffold for assembling the signaling complex (Greggio et al., 2017). LRRK2 is ubiquitary and controls processes even in neurons and glial cells. Dysregulated LRRK2 activity resulting from genetic mutations constitutes the primary cause of autosomal dominant PD. While the pathological impact of LRRK2 G2019S on EAAT2 has been explored (Iovino et al., 2022), its physiological role in the regulation of the expression and activity of membrane transporters has not been documented yet. Here we investigated the role of LRRK2 in modulating the expression and activity of selected excitatory and inhibitory NTTs by heterologous expression in *Xenopus laevis* oocytes coupled to a two‐electrode voltage clamp. The current amplitude and the biophysical properties in the presence and absence of LRRK2 were collected, and the results suggest a specific and crucial role of LRRK2 in the function and expression of EAAT2.

## 2 MATERIALS AND METHODS

### 2.1 cRNA preparation

Capped cRNAs coding for each protein were synthesized by in vitro transcription using T7 RNA polymerase from cDNAs linearized with restriction enzymes and then purified. Data about the plasmid vectors used for heterologous expression in *Xenopus laevis* oocytes are detailed in Table S1.

### 2.2 Heterologous protein expression and electrophysiological study in *Xenopus laevis* oocytes

All procedures performed in *Xenopus laevis* were approved by the Committee for the Animal Welfare of the University of Insubria (OPBA permit no. 06_20) and the Italian Ministry of Health (permit no. 449/2021‐PR).

*Xenopus laevis* oocytes were collected from adult females. The frogs were anesthetized in MS‐222 (tricaine methane sulfonate; Merck, Italy) 1 g/L solution in tap water, at pH 7.5 adjusted with sodium bicarbonate. The laparotomy was performed after sterilizing the abdomen with an antiseptic agent (Povidone‐iodine 0.8%) and the portions of the ovary removed. The total number of animals used for collecting the oocytes in this study was six, and all of them were bred in‐house. The number of animals used was initially estimated based on prior studies of a similar nature (Bhatt et al., 2024). As the collection of oocytes is considered a non‐invasive intervention, each animal went under surgery three times. The immersion in the anesthetic solution before the surgery provides an anesthetic effect that after the acute action lasts for hours post‐surgery, acting as a postoperative pain relief. After the surgery, the frogs were carefully monitored for 4 h and maintained in a small tank, then moved to a larger one for 24 h for the post‐operation recovery. The day after the surgery, the animals were moved back to a tank containing other frogs in post‐operative recovery for at least 6 months before the subsequent surgery.

Collected oocytes were treated with 1 mg/mL collagenase (collagenase type IA from *Clostridium histolyticum*, cat. no C0130 from Merck, Italy) in calcium‐free ND96 (96 mM NaCl, 2 mM KCl, 1 mM MgCl_2_, 5 mM 4‐(2‐hydroxyethyl)‐1‐piperazineethanesulfonic acid (HEPES) at pH 7.6) for at least an hour at 18°C. The healthy‐looking stage V and VI oocytes were selected and stored at 18°C in NDE solution (ND96 plus 2.5 mM pyruvate, 0.05 mg/mL gentamicin sulfate, and 1.8 mM CaCl_2_; Bhatt et al., 2022). In some experiments, the oocytes were purchased from Ecoocyte Bioscience GmbH, Germany.

The purified cRNAs were micro‐injected into the oocytes using a manual microinjection system (Drummond Scientific Company, Broomall, PA, USA). The concentrations of injected cRNAs encoding the protein of interest were 12.5 ng/50 nL for DAT, GAT1, and BGT‐1; and 25 ng/50 nL for EAAT2, EAAT1, GlyT1b, and GlyT2. The co‐expression with LRRK2 was performed by co‐injecting the same amount of the cRNA coding for the transporter plus 25 ng/50 nL of the cRNA coding for the kinase. All of the micro‐injected cRNAs were human analogs. These oocytes were stored in NDE at 18°C for the period of expression (typically 3–4 days), after which they were used for electrophysiological investigations.

The electrophysiological measurements were performed using a two‐electrode voltage clamp at controlled voltage conditions using an amplifier (Oocyte Clamp OC‐725 C or B; Warner Instruments, Hamden, CT, USA) and digitally recorded using the pCLAMP software (Version 11.2.2, Molecular Devices, San Jose CA, CA, USA). The two microelectrodes were filled with 3 M KCl, and bath electrodes were connected to the oocyte chamber via two agar bridges (3% agar in 3 M KCl). The composition of the external control solution (ND98 buffer) was: 98 mM NaCl, 1 mM MgCl_2_, 1.8 mM CaCl_2_, and 5 mM HEPES, adjusted to pH 7.6 with NaOH. The current signals were filtered at 0.1 kHz and sampled at 0.2 or 0.5 or 1 kHz. The experiments were performed using each substrate at a saturating concentration to record the transport‐associated current at a holding potential of −60 mV. The current–voltage (I‐V) relationship was obtained using a voltage‐jump protocol consisting of 9 square pulses (0.8 s long) ranging from −140 to +20 mV (at 20 mV increments). These traces were analyzed by subtracting the current recorded in the ND98 buffer from the current recorded in the presence of the substrate. The same analysis was also performed using NaNO_3_ and NaGluconate 98 mM, as replacement of NaCl from ND98 (see Figure 2).

The concentration‐response experiments were performed by exposing oocyte to increasing concentrations of substrate as indicated in each figure. The concentration‐response curves were fitted using the logistic growth Equation (1).

(1) $$y=\frac{A_{1}-A_{2}}{1+\left(x/x_{0}\right)}+A_{2}$$

### 2.3 Data analysis and statistics

All data analysis was performed using Clampfit 10.7 software (Molecular Devices, Sunnyvale, CA, USA). OriginPro 8.0 (OriginLab Corp., Northampton, MA, USA) and GraphPad Prism 8.0.2 (GraphPad Software, Boston, MA, USA) were used for statistical analysis and figure preparation.

**FIGURE 1:**
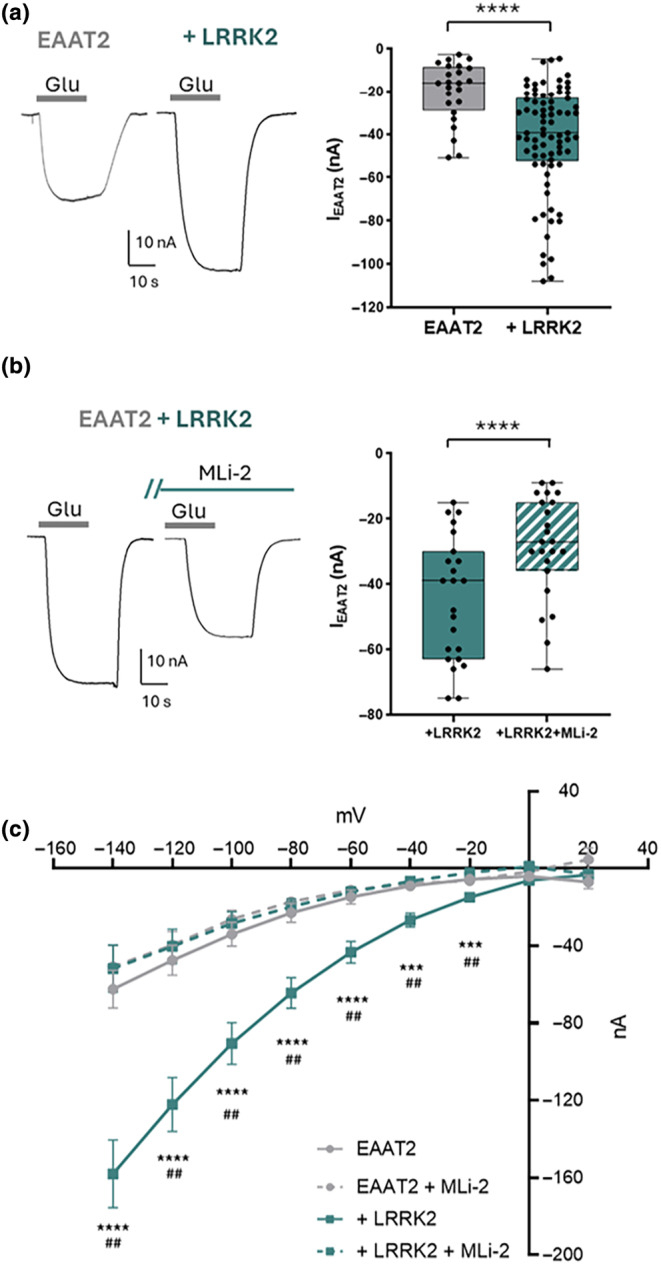
LRRK2 enhances EAAT2 transport current. (a) Representative traces of current amplitude elicited in oocytes expressing EAAT2 alone and with LRRK2 at the *V*_h_ = −60 mV, and the box plot represents the amplitude current upon glutamate application (1 mM; *n*/*N* = 24/4 and *n*/*N* = 78/12; Mann–Whitney test, *p* = 0.000011). (b) Representative traces of glutamate current elicited in oocytes co‐expressing EAAT2 + LRRK2 before and after MLi‐2 (200 nM) treatment at the *V*_h_ = −60 mV and the box plot of current amplitudes elicited in oocytes co‐expressing EAAT2 + LRRK2 before and after MLi‐2 (*n*/*N* = 23/6; Paired *t*‐test, *p* = 0.000005). The glutamate transport currents reported in the box plot are obtained by subtracting from the glutamate response the holding current recorded before the application of the substrate. (c) I–V relationship from −140 to +20 mV with 20 mV of increment (EAAT2: *n*/*N* = 19/3; EAAT2 + LRRK2: *n*/*N* = 13/3; EAAT2 after MLi‐2: *n*/*N* = 5/2; EAAT2 + LRRK2 after MLi‐2: *n*/*N* = 7/2. Unpaired *t*‐test; * EAAT2 vs. EAAT2 + LRRK2; # EAAT2 + LRRK2 vs. EAAT2 + LRRK2 after MLi‐2 (dotted line).

The Shapiro–Wilk test was used to assess the normality of the data. No tests for outliers were conducted. The statistical significance of the data was determined using the unpaired *t*‐test (Gaussian distribution) or Mann–Whitney test (non‐Gaussian distribution). A paired *t*‐test (Gaussian distribution) was applied for the comparison of two conditions of the same group (i.e., before and after MLi‐2 treatment (Figure 1)). Nonparametric multiple comparison was conducted using Kruskal–Wallis test followed by Dunn's test (i.e., single oocytes chemiluminescence analysis (Figure 4)). Levels of significance were defined as *p < 0.05; **p < 0.01; ***p < 0.001; ****p < 0.0001. The number of oocytes (*n*) obtained from a frog (*N*) used in each experiment is indicated as “n/N”.

### 2.4 Immunofluorescence

Oocytes expressing EAAT2 were fixed in ice‐cold 4% paraformaldehyde (PFA) in PBS at pH 7.5, for 15 min at 4°C, washed at room temperature (RT) in ND96 with mild agitation (5 min at RT, three times), included in Polyfreeze tissue freezing medium (Polysciences, Eppelheim) and frozen in liquid nitrogen. Oocytes were cryo‐sectioned into slices with a thickness of 10 μm using a Leica Biosystems Cryostat (Richmond, USA) and stored at −20°C. The oocyte slices underwent a 10‐min wash in PBS at RT and then incubated in a blocking solution (2% BSA (w/v), 0.1% Tween in PBS) for 45 min. Subsequently, the slices were incubated overnight at 4°C with the primary antibody.

A customized rabbit anti‐EAAT2 diluted (1:50) in the blocking buffer was kindly provided by Dr. Perego (Iovino et al., 2022; Perego et al., 2000). Then sections were washed in PBS and incubated with the secondary antibody rabbit DyLight 488 (1:1000 in blocking buffer; cat. no A78956 from ThermoFisher) for an hour and washed in PBS three times, the sections were mounted using Mowiol, and the images were acquired with ZOE Fluorescent Cell Imager (Bio‐rad).

### 2.5 Single oocyte chemiluminescence (SOC)

After fixation, as reported for immunofluorescence, the oocytes were subjected to a one‐hour incubation in a blocking solution (1% BSA in ND96) and then incubated for 2 h with primary antibody guinea pig anti‐GLT1 (1:1000 in blocking solution; cat. no GC‐022‐GP from Amolone Labs) at 4°C, followed by a 5‐min wash repeated six times. The oocytes were then transferred RT and maintained for an hour in peroxidase‐conjugated anti‐guinea pig (1:20.000 in blocking solution; cat. no A‐21450 from ThermoFisher), with rinsing for 3 min repeated six times in 1% BSA in ND96 and 3 min repeated six times in ND96 alone. Finally, each oocyte was transferred to a 96‐well plate containing 50 μL of SuperSignal West Femto (Pierce, ThermoFisher Scientific). The chemiluminescent signal was quantified using a Tecan Infinity 200 microplate reader (Tecan). The plate was read within 5 min after the transfer of the first oocyte.

## 3 RESULTS

### 3.1 The kinase LRRK2 enhances EAAT2 transport activity

To evaluate the physiological role of LRRK2 on EAAT2, human glutamate transporter EAAT2 mRNA was injected alone and co‐injected with human LRRK2 wild‐type mRNA into *Xenopus laevis* oocytes. The current was recorded in the presence of glutamate (1 mM) at −60 mV. The representative traces of oocytes expressing EAAT2 and EAAT2 + LRRK2, and the mean amplitude of the glutamate‐associated currents (I_EAAT2_) are reported in Figure 1a. Oocytes expressing EAAT2 and LRRK2 gave rise to an inward current (−41.9 ± 2.7 nA; n/*N* = 78/12) significantly higher compared to that obtained from oocytes injected with EAAT2 alone (−20.05 ± 2.8 nA; n/*N* = 24/4; Mann–Whitney test, *p* = 0.000011, U = 399.5; Figure 1a). To confirm the effect of LRRK2 on EAAT2 transport current, LRRK2 activity was blocked using MLi‐2, a potent and selective LRRK2 inhibitor that de‐phosphorylates Ser935 in LRRK2 (Fell et al., 2015). The glutamate transport current was measured on the same oocytes before and after the MLi‐2 incubation (200 nM, 90 min). The representative traces (Figure 1b) show that the glutamate‐evoked current in oocytes co‐expressing EAAT2 + LRRK2 is reduced after MLi‐2 treatment; the data in the boxplot statistically confirmed the effect (−44.5 ± 3.9 nA vs. −28.6 ± 3.3 nA before and after MLi‐2 treatment respectively; Paired Student *t*‐test, *p* = 0.000005, t = 5.97, df = 22). When the LRRK2 is blocked, I_EAAT2_ becomes similar to that recorded in oocytes expressing EAAT2 alone.

The current–voltage relationships (Figure 1c) were obtained by plotting the mean of the transport‐associated current from voltage‐steps protocol. The data at the negative membrane potentials confirmed the results obtained by the tape recording at *V*_h_ = −60 mV. The glutamate transport‐associated current is significantly increased when EAAT2 is co‐expressed with LRRK2, whereas after MLi‐2 treatment, EAAT2 transport activity is significantly impaired (Table S2). The overlapping of current amplitudes in the last condition with the oocytes expressing the transporter alone provides additional evidence for the role of LRRK2 in sustaining the functionality of the glutamate transporter. Moreover, MLi‐2 treatment did not change the glutamate transporter current recorded in oocytes expressing EAAT2 alone (Figure 1c).

### 3.2 The kinase LRRK2 does not modify the glutamate‐induced anion conductance of EAAT2

EAAT2 is an anion‐selective ion channel like other EAATs, although the anion current is only a small component of the overall transporter‐associated current amplitude (Torres‐Salazar & Fahlke, 2007). To understand whether LRRK2 influences the anionic component of EAAT2, we investigated the glutamate transport current in the presence and absence of the kinase. The voltage‐step protocol was applied in the presence of solutions with three different anionic compositions with and without glutamate. The NaGluconate buffer solution was made with a reduced anionic component (to 7 mM of chloride) by substituting the NaCl with NaGluconate. The NaNO_3_ buffer solution was used to have an anionic component with a higher anionic driving force than the NaCl one (Kolen et al., 2020). The glutamate transport current in the three solutions was obtained by subtracting the current recorded in the corresponding anionic buffer.

**FIGURE 2:**
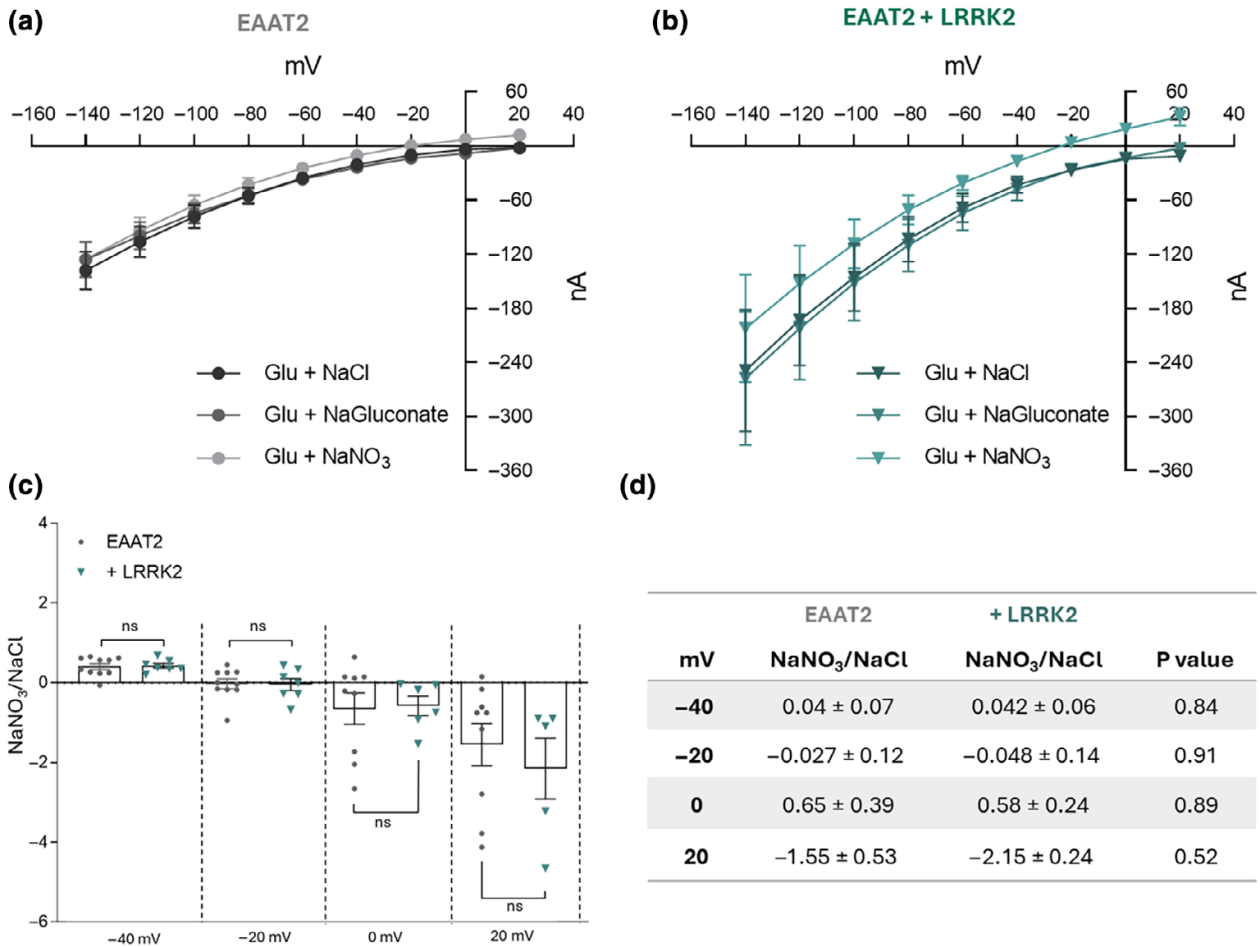
LRRK2 alters the EAAT2 transport current without affecting the anionic component. (a, b) I–V relationship from −140 to +20 mV with 20 mV of increment in the presence of chloride (NaCl), nitrate (NaNO_3_), and gluconate (NaGluconate) solutions in oocytes injected with EAAT2 alone (Glu + NaCl: *n*/*N* = 12/2; Glu + NaGluconate: *n*/*N* = 10/2; Glu + NaNO_3_: *n*/*N* = 11/2;) and co‐injected with EAAT2 + LRRK2 (Glu + NaCl: *n*/*N* = 8/2; Glu + NaGluconate: *n*/*N* = 8/2; Glu + NaNO_3_: *n*/*N* = 8/2). (c) Ratio of glutamate current amplitude recorded for NaNO_3_/NaCl solutions in oocytes expressing EAAT2 alone (gray dots) and co‐expressing EAAT2 + LRRK2 (green triangles) at following voltages: −40, −20, 0, and 20 mV. All currents were recorded at *V*_h_ = −60 mV. (d) Table of the ratio NaNO_3_/NaCl glutamate current in oocytes expressing EAAT2 with and without LRRK2. All values are shown as mean ± SEM, *p*‐values were obtained using Unpaired student *t*‐test.

In Figure 2, the current–voltage relationship shows that the kinase does not change the amplitude of the anionic component of the current (Figure 2a,b). To better highlight this aspect, we reported the ratio between the glutamate anion component (current recorded in the presence of NaNO_3_ buffer solution) and glutamate transport current (in the presence of standard buffer solution) from −40 to 20 mV. The histogram and table show that there are no significant differences between the two conditions (Figure 2c,d).

### 3.3 The kinase LRRK2 changes EAAT2 transport parameters

**FIGURE 3:**
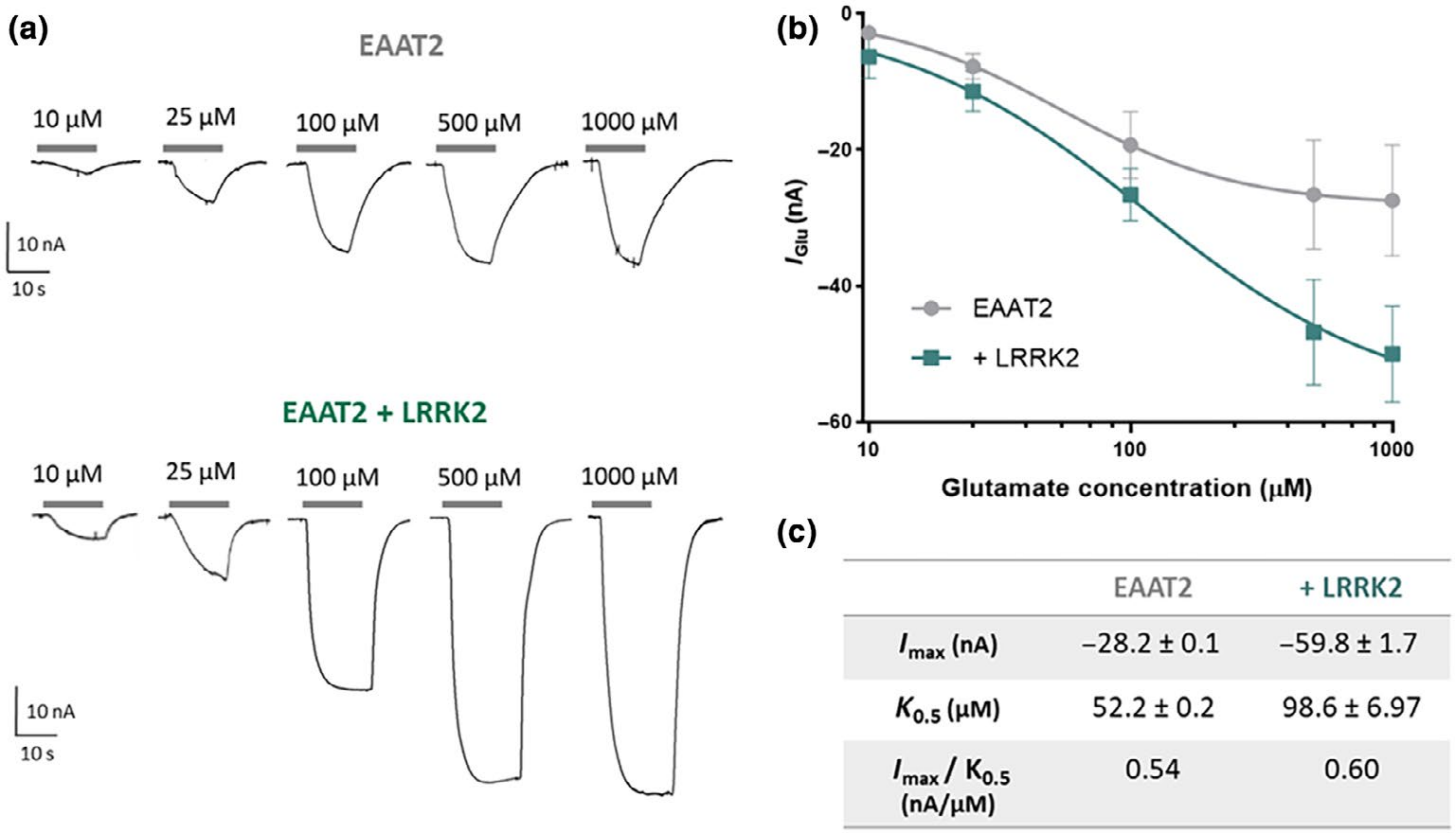
EAAT2 kinetic parameters in the presence of LRRK2. (a) Representative traces of concentration‐response in EAAT2. The transport current elicited by increasing glutamate concentration perfused (10, 25, 100, 500 μM, 1 mM) in oocytes expressing EAAT2 alone (top) and with LRRK2 (bottom). (b) Concentration‐response curve fitting with the logistic growth equation (EAAT2: *n*/*N* = 6/3; EAAT2 + LRRK2: *n*/*N* = 6/3). (c) Tabular presentation of the kinetic parameters *I*_max_, *K*_0.5_, and transport efficiency.

In the concentration‐response experiment of EAAT2, the glutamate transport current elicited by increasing concentrations of substrate (from 10 μM to 1 mM) was measured in the presence and absence of LRRK2. The representative traces in Figure 3a display the increased current in the presence of LRRK2. Data were fitted using equation 1 and the obtained kinetic parameters and transport efficiency of EAAT2 alone and in the presence of LRRK2 were reported in Figure 3c.

The comparison of the parameters collected shows that LRRK2 decreases the EAAT2 glutamate affinity (Parametric Student's *t*‐test, *p* = 0.04, *t* = 2.41, df = 9; Figure 3b) but increases maximal transport current (*I*_max_), resulting in unaltered transport efficiency (*I*_max_/*K*_0.5_).

### 3.4 LRRK2 is required for EAAT2 membrane localization

**FIGURE 4:**
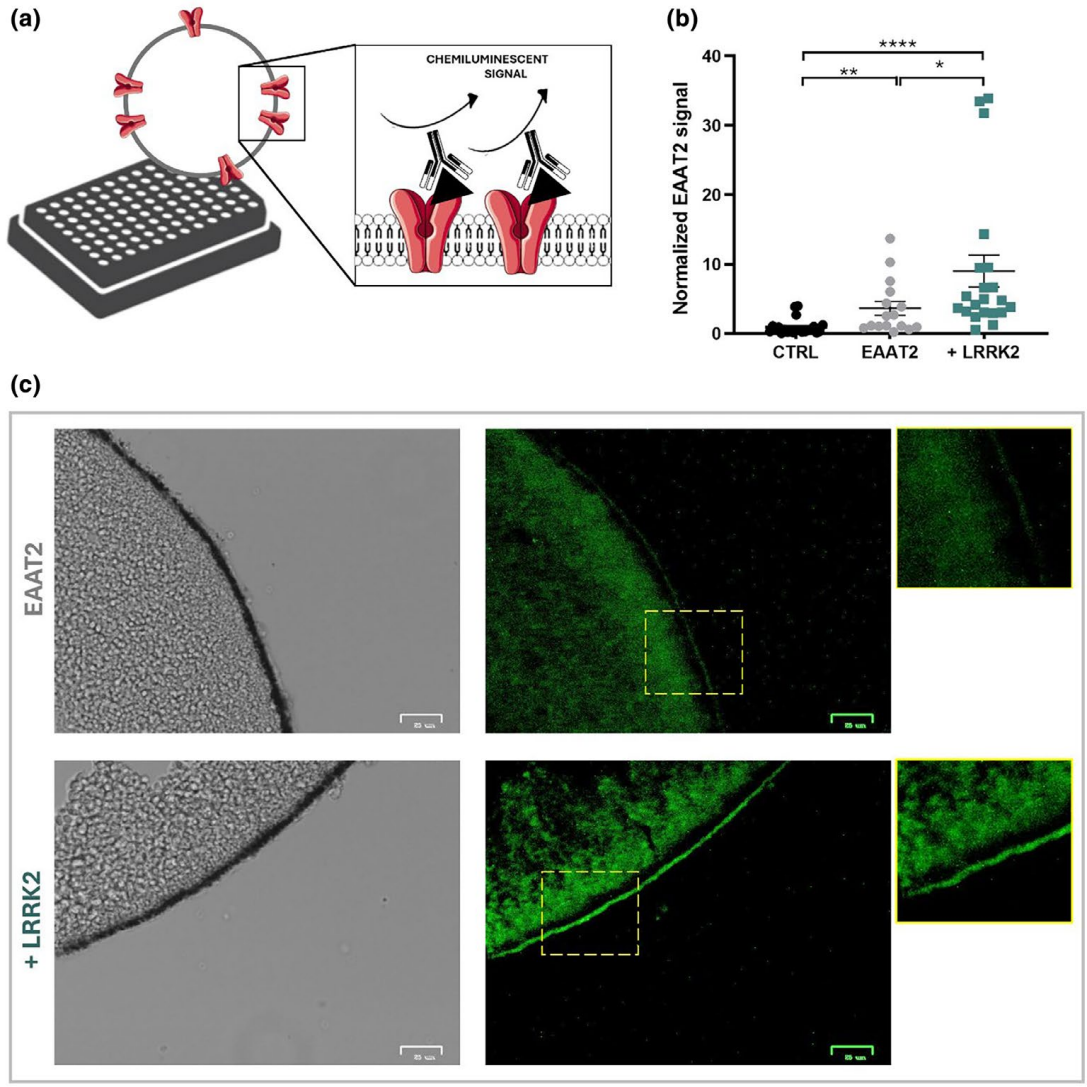
LRRK2 prompts EAAT2 membrane localization. (a) Schematic outline of SOC protocol. The antibody against EAAT2 detects the amount of transporter at the plasma membrane. (b) Scatter plot shows the quantification of EAAT2 chemiluminescent signal in oocytes injected with EAAT2 alone (n/*N* = 16/3) and co‐injected with LRRK2 (n/*N* = 21/3). Data were normalized on non‐injected oocytes used as internal control (n/*N* = 22/3). (c) Representative bright‐field (left column) or fluorescence (ex480‐517; right column) images of oocyte slices expressing EAAT2 alone (green signal) or with LRRK2; scale bar 25 μm.

The kinetic parameters of EAAT2 suggest that in the presence of LRRK2, the number of active transporters is increased. To investigate the relationship between LRRK2 and EAAT2, SOC was used to quantify the amount of transport proteins expressed (see the schematic outline in Figure 4a). The quantitative analysis of the result showed a higher presence of glutamate transporters in oocytes co‐expressing EAAT2 with LRRK2 than in those injected with EAAT2 alone (Mann–Whitney test, *p* = 0.0224, U = 94). All results were normalized using the non‐injected oocytes as internal controls (Dunn's test following a Kruskal–Wallis test, EAAT2 vs. ctrl *p* = 0.0068; EAAT2 + LRRK2 vs. ctrl *p* = 0.0001; Figure 4b). We also performed immunofluorescence experiments on oocyte slices that showed a different intensity of the green signal both at the plasma membrane and cytoplasm in the presence and absence of the kinase. Although immunofluorescence signals cannot be exactly quantified in this context, both methods support the idea that the amount of transport protein is lower in the absence of the LRRK2 (Figure 4c).

### 3.5 The kinase LRRK2 does not affect the transport activity of other NTTs

**FIGURE 5:**
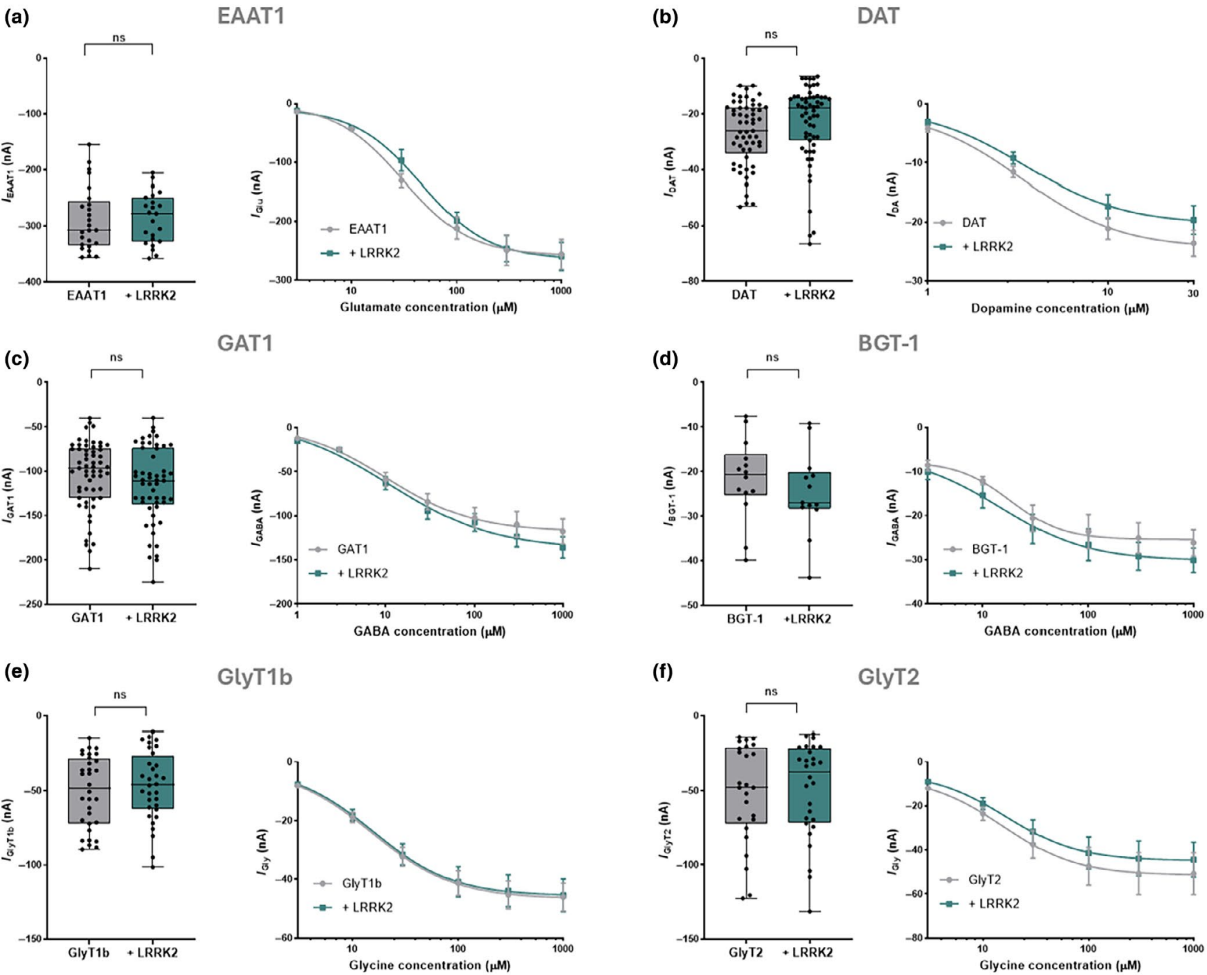
(a) (Left) The box plot of the mean of the glutamate (1 mM) transport‐associated current amplitudes elicited in oocytes expressing EAAT1 (*n*/*N* = 25/2) and co‐expressing EAAT1 and LRRK2 (*n*/*N* = 23/2). (Right) Concentration‐response curve fitting from oocytes expressing EAAT1 (*n*/*N* = 8/2) and co‐expressing EAAT1 and LRRK2 (*n*/*N* = 4/2) exposed to increasing concentrations of glutamate. (b) (Left) The box plot of the mean of the amplitudes of the dopamine (30 μM) transport‐associated current elicited in oocytes expressing DAT (*n*/*N* = 57/2) and co‐expressing DAT and LRRK2 (*n*/*N* = 59/2). (Right) Concentration‐response curve fitting from oocytes expressing DAT (*n*/*N* = 13/2) and co‐expressing DAT and LRRK2 (*n*/*N* = 12/2). (c, d) (Left) The box plot represents the mean of the amplitudes of the GABA (1 mM) transport‐associated current elicited in oocytes expressing GAT1 (*n*/*N* = 59/3) and co‐expressing GAT1 and LRRK2 (*n*/*N* = 53/3) or BGT‐1 (*n*/*N* = 14/2) and co‐expressing BGT‐1 and LRRK2 (*n*/*N* = 13/2). (Right) Concentration‐response curve fitting from oocytes expressing from oocytes expressing GAT1 (*n*/*N* = 11/2) and co‐expressing GAT1 and LRRK2 (*n*/*N* = 11/2) or BGT‐1 (*n*/*N* = 7/2) and co‐expressing BGT‐1 and LRRK2 (*n*/*N* = 7/2). (e, f) (Left) The box plot represents the mean of the amplitudes of the glycine (1 mM) transport‐associated current elicited in oocytes expressing GlyT1b and co‐expressing GlyT1b and LRRK2 (*n*/*N* = 32/2) or GlyT2 (*n*/*N* = 25/2) and co‐expressing GlyT2 and LRRK2 (*n*/*N* = 28/2). (Right) Concentration‐response curve fitting from oocytes expressing GlyT1b and co‐expressing GlyT1b and LRRK2 (*n*/*N* = 19/2) or GlyT2 (*n*/*N* = 13/2) and co‐expressing GlyT2 and LRRK2 (*n*/*N* = 16/2). All currents were collected at the *V*_h_ = −60 mV. All groups were statistically analyzed using an unpaired Student *t*‐test. Concentration‐response curves were fitted using the logistic growth fitting model.

As a result of the significant impact of LRRK2 on EAAT2 transport expression and function, we investigated the specificity of the modulation by injecting *Xenopus laevis* oocytes with cRNA of other NTTs in the presence or the absence of the kinase. We selected different members of SLC1 and SLC6 expressed in neurons and glial cells: EAAT1 (SLC1A3), DAT (SLC6A3), GAT1 (SLC6A1), BGT‐1 (SLC6A12), GlyT1b (SLC6A9), and GlyT2 (SLC6A5). The transport current amplitude and the concentration‐response relationship were measured as for EAAT2 in Figures 1a and 3. The mean of the recorded current for each transporter in the presence and absence of the kinase was measured and reported as box plots. The current collected at increasing doses of substrates was reported in concentration‐response curves. All the graphs and details of kinetic parameters from the fitting of concentration‐response are reported in Figure 5 and Table 1.

**TABLE 1:** Kinetic parameters collected for each tested NTTs with and without LRRK2.

	*I*_max_ (nA)	*K*_0.5_ (μM)	Hill's coefficient (*n*)	*I*_max_/K_0.5_ (nA/μM)
EAAT1	−252.05 ± 7.41	30.54 ± 1.92	1.57 ± 0.09	8.25
EAAT1 + LRRK2	−270.16 ± 10.88	43.72 ± 4.71	1.16 ± 0.08	6.17
DAT	−24.94 ± 0.87	3.42 ± 0.23	1.43 ± 0.1	7.28
DAT + LRRK2	−20.86 ± 0.78	3.56 ± 0.25	1.41 ± 0.09	5.85
GAT1	−113.4 ± 4.12	11.54 ± 1.27	1.12 ± 0.12	9.82
GAT1 + LRRK2	−125.07 ± 7.57	13.08 ± 2.63	1.28 ± 0.24	9.55
BGT‐1	−25.45 ± 0.58	18.94 ± 1.59	1.86 ± 0.26	1.34
BGT‐1 + LRRK2	−30.22 ± 0.67	15.42 ± 3.11	1.06 ± 0.21	1.96
GlyT1b	−46.52 ± 0.35	15.54 ± 0.36	1.12 ± 0.04	2.99
GlyT1b + LRRK2	−45.54 ± 0.22	16.01 ± 0.22	1.15 ± 0.02	2.84
GlyT2	−51.54 ± 0.42	15.04 ± 0.35	1.18 ± 0.05	3.42
GlyT2 + LRRK2	−44.94 ± 0.41	16.20 ± 0.41	1.21 ± 0.05	2.77

*Note*: The maximal current (*I*_max_), the apparent affinity (*K*_0.5_), Hill's coefficient (*n*) are reported as the mean value ± SEM, and the transport efficiency (*I*_max_/*K*_0.5_) was calculated for each group. All raw data were collected at *V*_h_ = −60 mV and fitted with a logistic growth equation.

The transport current in oocytes expressing EAAT1 alone, as expected, at –60 mV was larger than the current elicited by EAAT2 (Wadiche et al., 1995). However, the co‐expression with LRRK2 did not alter the electrophysiological properties of EAAT1 (Figure 5a and Table 1). Similarly, the results showed that the DAT activity was not altered by the presence of LRRK2 (Figure 5b).

Moreover, we investigated the membrane inhibitory neurotransmitter transporters: GAT1, BGT‐1, GlyT1b, and GlyT2, by expressing and testing them alone and in the presence of the kinase. Remarkably, all of them did not show any changes in expression in terms of current amplitude and kinetic parameters in the presence of LRRK2 (Figure 5c–f, and Table 1).

The effect of the mutated LRRK2 G2019S was also tested on all the above‐mentioned transporters. No alterations in their electrophysiological properties were observed, as detailed in the Table S3. The raw data obtained for each experimental set and statistical test are reported in Tables S4 and S5.

## 4 DISCUSSION

The activity and expression of membrane transporters are regulated by protein‐specific mechanisms. These mechanisms encompass the regulation of transporter expression and distribution between the cell surface and intracellular compartments, as well as the modulation of substrate affinity, maximal velocity, maximal current, rate, and efficiency of transport (Moritz et al., 2013; Ryan et al., 2021). However, our understanding of the specific players involved in these regulatory processes remains incomplete. Such modulations must be quite specific, considering the reported dynamic regulation on a time scale of seconds/minutes and the transient changes in membrane potential that modify the activity of transporters at the plasma membrane (Ryan et al., 2021; Zahniser & Doolen, 2001).

The present study unveils a functional interplay between LRRK2 and glial glutamate transporter EAAT2. Our findings demonstrate the role of this kinase on EAAT2 function and expression at the plasma membrane. Crucially, the data emphasizes LRRK2 as a critical factor for EAAT2 membrane localization, with a regulatory mechanism distinct from other NTTs tested here, i.e., EAAT1, DAT, GAT1, BGT‐1, GlyT1b, and GlyT2. Consequently, the proper functioning of LRRK2 is crucial for maintaining glutamate homeostasis.

Indeed, the number of glutamate transporters, particularly EAAT2, is essential for the clearance of the NT from the synaptic cleft and avoiding its spilling over. Even if the turnover rate of NTTs is slow (for EAAT2 in the range of 19 s^−1^ (Kabakov & Rosenberg, 2015)), it is the high number of the transporter expressed at the synaptic level that strictly controls the amount of NT and consequently the receptor activity (Koch & Larsson, 2005; Lester et al., 1996).

The observed increase in glutamate transport current in the presence of LRRK2 highlights the role of EAAT2 in rapidly uptaking extracellular glutamate in the glial cells to regulate glutamatergic signaling. Conversely, the absence of LRRK2 kinase markedly reduces the transporter function, as evidenced by a significant decrease in the transport current and expression assay.

Furthermore, pharmacologically inhibiting the LRRK2 activity by treating the oocytes expressing EAAT2 with MLi‐2, decreases the glutamate transport current amplitude like that recorded in the absence of the kinase. This experiment confirmed the role of LRRK2 on EAAT2 function, validating their physiological functional relationship. Moreover, the individual components of the total EAAT2‐mediated glutamate transport current are influenced in the same way by the presence of the kinase. The increase of the glutamate‐induced anion conductance is proportional to the increase of the glutamate current. The ratio of NaNO_3_/NaCl‐induced glutamate currents in the presence or absence of the kinase is identical. This data supports the role of LRRK2 in increasing the number of functional transporters.

We used single oocyte chemiluminescence analysis to compare the amount of EAAT2 protein at the plasma membrane with and without LRRK2. Notably, the signal detected in the absence of the kinase was significantly lower than observed in its presence, indicating the pivotal role of LRRK2 in facilitating EAAT2 localization at the plasma membrane. These findings were qualitatively validated using immunofluorescence staining on oocyte slices. A brighter fluorescence signal at the plasma membrane was visible when EAAT2 was co‐expressed with LRRK2. Thus, our data show that LRRK2 enhances the plasma membrane localization of EAAT2, increasing the transporter availability and glutamate clearance activity. Moreover, LRRK2 enhances both the apparent affinity for glutamate and the maximal current of EAAT2 without altering the transporter efficiency, which remains similar in both experimental conditions. All data collected in the presence of LRRK2 supports the idea that the increased activity is correlated with the number of functional transporters at the plasma membrane, rather than with a modifying substrate interaction or cycling rates. Interestingly, the ratio of *I*_max,EAAT2_ to *I*_max,EAAT2+LRRK2_ is 0.47, which closely equals the ratio of the expression level revealed by single oocyte chemiluminescence analysis (AU_EAAT2_/AU_EAAT2+LRRK2_ = 0.40). These ratios indicate a correlation between the expression at the plasma membrane of the protein and its function in the presence and absence of LRRK2. Notably, our results do not provide insight into the specific stage of the LRRK2 action, whether translation, protein assembly, or membrane trafficking. However, our previous evidence demonstrated that the pathological mutant G2019S variant of LRRK2, implicated in the common form of familial Parkinsonism, severely affects EAAT2 expression (Iovino et al., 2022). This study demonstrated that EAAT2 trafficking is disrupted by the pathogenic variant G2019S of LRRK2. Both human brains and experimental animal models showed significantly decreased levels of EAAT2 protein because of the hyperphosphorylated activity of the mutated kinase. These findings and our results highlight the critical role of LRRK2 in EAAT2 biology in both physiological and pathological conditions, supporting the hypothesis that EAAT2 and the LRRK2 kinase are crucial factors in maintaining glutamate homeostasis in CNS. *Xenopus laevis* oocytes are an appropriate tool for investigating the in vitro function of membrane proteins but are not the ideal system for defining the intracellular molecular mechanism. Consequently, the data about LRRK2 modulation on EAAT2 reported here is limited to the functional observation and lacks precise quantification of the amount of the protein in the plasma membrane and the cytoplasm, as a result of the unfeasibility of precise quantification of the protein in the different cellular compartments (Jorgensen et al., 2016). However, our findings strongly suggest that LRRK2 increases the amount of functional EAAT2 (Figures 1, 3) and is involved in membrane localization (Figure S1). Remarkably, LRRK2 activity selectively affects EAAT2, with no effect on EAAT1 or other tested neurotransmitter transporters, highlighting the specificity of the LRRK2‐EAAT2 interaction.

Recent research on the modulation of DAT, well summarized by Bu et al., reported the impact of LRRK2 on DAT function (Bu et al., 2021). PET imaging in human and animal studies suggests early DAT dysfunction induced by LRRK2 G2019S mutants, with elevated DA turnover and reduced DAT levels. Overexpression of mutant LRRK2 impairs DAT‐mediated DA reuptake in animal models. These effects could be related to the role of LRRK2 in intracellular membrane trafficking, mediated by Rab protein phosphorylation. Data in the literature show that the LRRK2 G2019S mutation leads to progressive DAT dysfunction. Pharmacological analyses support impaired DAT function in mutant LRRK2 transgenic rats, with behavioral changes observed in aged animals but not in young ones. Remarkably, temporal but not constitutive overexpression of mutant LRRK2 induces phenotypes, suggesting indirect loss to DAT activity. The possibility that the effect of LRRK2 on DAT is indirect is further supported by in vitro analysis reported by Zhou et al. (2011). In that work, Zhou et al. did not detect any direct interaction and effect of mutant or wild‐type LRRK2 with DAT, when overexpressed in the neuroblastoma cells SH‐SY5Y as a model of dopaminergic neurons. These data perfectly agree with our result in *Xenopus laevis* oocytes expressing DAT with and without LRRK2 wild type and G2019S mutants. One of the possible players involved in the indirect effect on DAT is suggested by Bu et al., where it is reported that VPS35 and LRRK2 functionally interact to regulate DAT function and striatal dopamine transmission (Bu et al., 2023). Apart from its relationship with EAAT2, LRRK2 shows no action on any of the NTTs studied here, including DAT, in the *Xenopus laevis* oocytes system. An unresolved aspect concerns the potential pathway directly connecting EAAT2 and LRRK2 under physiological conditions. Iovino et al. propose that LRRK2 G2019S might influence EAAT2 turnover from the plasma membrane by impacting the cellular degradative system (Iovino et al., 2022). This mechanism involves the accumulation of transporters within the Rab4‐positive compartment because of the hyperphosphorylated activity of pathogenic LRRK2. However, the pathological and physiological actions of LRRK2 may engage distinct pathways, particularly given the multifaceted nature of this protein. Interestingly, the work of B. M. Roberts et al., about the regulation of striatal GABA‐DA relations suggests a role of the striatal GATs and astrocytes in the maladaptive plasticity typical of early parkinsonism (Roberts et al., 2020). The effect could be related to a new indirect action of the pathogenic LRRK2 like what was reported in the literature (Bu et al., 2023; Cataldi et al., 2018).

Our research shows that in EAAT1, GAT1, BGT‐1, GlyT1b, and GlyT2, the maximal currents, substrate affinity, and expression are unaltered by the co‐expression of LRRK2 (wild type or mutated), which raises new questions about the mechanism of its action. The absence of direct action on DAT and on the other NTTs does not exclude the possibility that, as already shown for DAT, the pathological mutants of LRRK2 could have an indirect action (Bu et al., 2023) differently from the direct action on EAAT2 (Iovino et al., 2022).

In conclusion, our study, for the first time, demonstrates the physiological importance of LRRK2 in EAAT2 function, highlighting this regulatory mechanism and identifying a potential target for therapeutic interventions. Our findings suggest that pharmacological strategies aimed at enhancing transporter activity via LRRK2 could ameliorate pathological conditions not only in Parkinson's disease but also across a spectrum of neurodevelopmental and neurodegenerative disorders. In these conditions, where hyperexcitability can lead to excitotoxicity because of compromised glutamate clearance, targeting LRRK2 for EAAT2 regulation may offer novel therapeutic opportunities.

## AUTHOR CONTRIBUTIONS

**Angela Di Iacovo:** Conceptualization; investigation; writing – original draft; data curation; methodology; writing – review and editing. **Chiara D'Agostino:** Investigation; methodology; data curation; writing – original draft. **Manan Bhatt:** Conceptualization; investigation; writing – original draft; writing – review and editing; data curation; methodology. **Tiziana Romanazzi:** Conceptualization; investigation; writing – original draft; methodology. **Stefano Giovannardi:** Investigation; methodology. **Raffaella Cinquetti:** Methodology; investigation. **Cristina Roseti:** Conceptualization; investigation; writing – review and editing; supervision; methodology. **Elena Bossi:** Conceptualization; investigation; funding acquisition; writing – original draft; writing – review and editing; validation; supervision; resources; formal analysis.

## CONFLICT OF INTEREST STATEMENT

The authors declare that the research was conducted without any commercial or financial relationships that could be construed as a potential conflict of interest.

### PEER REVIEW

The peer review history for this article is available at https://www.webofscience.com/api/gateway/wos/peer‐review/10.1111/jnc.16265.

## Supporting information


Data S1.

## Data Availability

The data that support the findings of this study are available from the corresponding author upon reasonable request.
