# Supplementary material for: The kinase LRRK2 is required for the physiological function and expression of the glial glutamate transporter EAAT2 (SLC1A2)
Source: J Neurochem. 2024 Dec 10;169(1):e16265. doi: 10.1111/jnc.16265 (PMC11629453; doi:10.1111/jnc.16265)
Supplement: Supplementary file 1 — Data S1. [file JNC-169-0-s001.zip › JNC-2024-0242.R2_Supplementary tables NTT + LRRK2_R3.pdf]

The kinase LRRK2 is required for the physiological function and expression of the glial glutamate transporter EAAT2 (SLC1A2).

Angela Di Iacovo [1,3], Chiara D'Agostino[1,2,3], Manan Bhatt [1,3], Tiziana Romanazzi[1,3], Stefano Giovannardi[1], Raffaella Cinquetti [1], Cristina Roseti [1,3], Elena Bossi [1,3]

Affiliation

[1]Department of Biotechnology and Life Sciences, Laboratory of Cellular and Molecular Physiology, University of Insubria, Varese, Italy.

[2] PhD School of Experimental and Translational Medicine, University of Insubria, Varese, Italy.

[3]Centre for Neuroscience, University of Insubria, Varese, Italy.

Corresponding Author Elena Bossi- email: elena.bossi@uninsubria.it

Supplementary Document

The action of MLi-2, an inhibitor of LRRK2 on the glutamate-induced current

The mean current amplitude elicited in the presence of glutamate in the EAAT2+LRRK2 and EAAT2 alone expressing oocytes, before and after MLi-2 treatment and after its removal are reported in Figure S1. After 90 minutes of treatment with MLi-2 the current amplitude only of the oocytes expressing EAAT2 with LRRK2 is reduced and became similar to that recorded from oocytes expressing the EAAT2 alone (Unpaired t-test, p=0.37). After MLi-2 treatment, the oocytes were moved on a plate in medium solution (NDE) for 90 minutes to remove the inhibitor. The current measured after the recovery (white column) returned to the amplitude recorded at the beginning of the experiments (Paired t-test, p=0.56). Whereas the oocytes expressing EAAT2 alone were unaffected by the treatment of MLi-2. As a control, we incubated the oocytes expressing EAAT2 plus LRRK2, and EAAT2 alone in NDE to mimic the timing of MLi-2 treatment. No significant alteration in the current amplitude was visible in both experimental groups. Notably, the time (90 minutes) required for the current amplitude reduction suggests membrane transporter recycling rather than transporter degradation and de novo synthesis (Soreq & Seidman, 1992).

Figure S1

a.

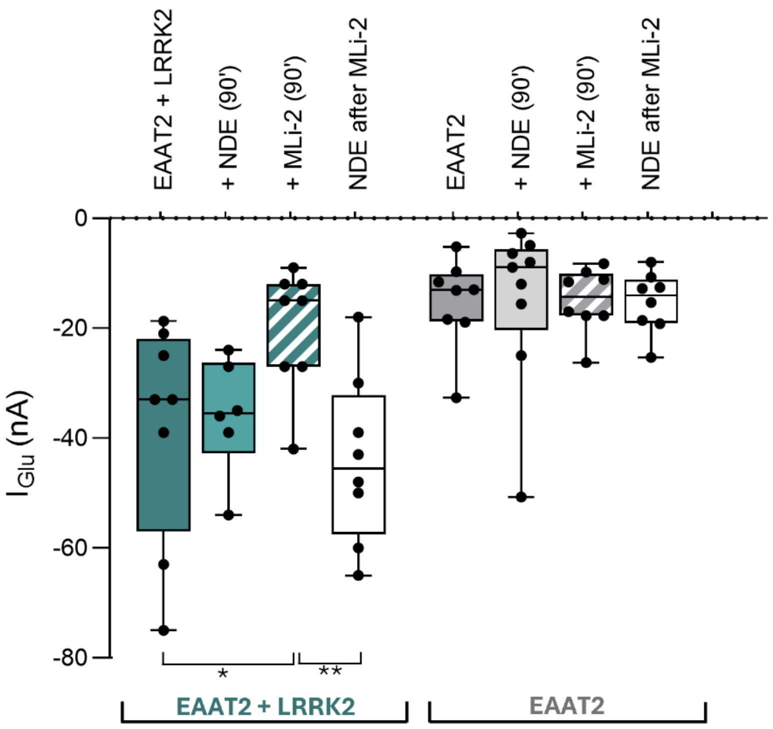

b.

	P value
EAAT2 + LRRK2 vs + NDE (90')	0.901
EAAT2 + LRRK2 vs + MLi-2 (90')	0.015
EAAT2 + LRRK2 vs + NDE after MLi-2 (90')	0.569
EAAT2 + LRRK2 + MLi-2 (90') vs + NDE after MLi-2 (90')	0.003
EAAT2 + LRRK2 + MLi-2 (90') vs + NDE (90')	0.158
EAAT2 + LRRK2 + NDE (90') vs + NDE after MLi-2 (90')	0.118
EAAT2 + vs + NDE (90')	0.957
EAAT2 vs + MLi-2 (90')	0.769
EAAT2 vs + MLi-2 (90') + NDE (90')	0.995

Fig S1. (a.) The box plot shows the glutamate transport current amplitude recorded in oocytes expressing EAAT2+LRRK2 (on left) and EAAT2 alone (on right). Conditions: before (dark green and dark grey columns respectively) and after the incubation in LRRK2 inhibitor MLi-2 for 90 min (stripped columns; EAAT2+LRRK2 pre and post MLi-2: paired t-test p=0.015, t=3.171, df=7, n/N=8/1) or incubated in NDE solution without MLi-2 for 90 min (light green, n/N=6/1, and light grey, n/N=9/1); and after removal of MLi-2 by letting the oocytes for 90 minutes in NDE (white columns; EAAT2+LRRK2+MLi-2 vs EAAT2+LRRK2 after wash: paired t-test p=0.003, t=4,314, df=7, n/N=8/1). In each single experimental group, where significance is not mentioned, it is considered to be not significant. (**b.**) Statistical analysis (paired t-test) on raw data obtained from amplitudes of the substrate transport associated-current, comparing oocytes of each group (EAAT2 + LRRK2; EAAT2) in each tested condition as shown in box-plot in (a.).

Table S1

Protein	Gene	Accession Number	Organism	Vector	REase used for linearization
**EAAT2**	SLC6A2	NM_004171.4	Homo sapiens	pCDNA3	Hind III
**EAAT1**	SLC1A3	NM_004172.5	Homo sapiens	pCDNA3	Sma I
**DAT**	SLC6A3	NM_010020.3	Mus Musculus	pNBI	Not I
**GAT1**	SLC6A1	NM_003042	Homo sapiens	pEXPslm	Nhe I
**BGT-1**	SLC6A12	NM_003044	Homo sapiens	pEXPslm	Nhe I
**GlyT1b**	SLC6A9	NM_006934.4	Homo sapiens	pCDNA3	Sma I
**GlyT2**	SLC6A5	NM_004211.5	Homo sapiens	pCDNA	Nde I
**LRRK2**	LRRK2	NM_198578.4	Homo sapiens	pDESTN-SF-TAP	Sma I

Table S1. List of transporter gene and vector used for heterologous expression in *X. laevis* oocytes; (REase: restriction enzyme).
Plasmids were provided as follows: EAAT1 and LRRK2 by Dr. Laura Civiero (University of Padua); GAT1 and BGT-1 by Petrine Wellendorph (University of Copenhagen); EAAT1 by Christoph Fahlke (Institute of Complex Systems, Zelluläre Biophysik Forschungszentrum Jülich); DAT by Urlik Gether (University of Copenhagen); GlyT1b and GlyT2 by Aurelio Galli (University of Alabama).

Table S2

Voltages (mV)	P value (EAAT2 vs EAAT2 + LRRK2)	P value (EAAT2 + LRRK2 vs EAAT2 + LRRK2 after MLi-2)
**-140**	0.000018	0.0010
**-120**	0.000023	0.0016
**-100**	0.000034	0.0023
**-80**	0.000049	0.0037
**-60**	0.000093	0.0058
**-40**	0.000164	0.0080
**-20**	0.000190	0.0041
**0**	0.32	0.00038
**20**	0.38	0.18

Tab S2. Statistical analysis (unpaired t-test) on raw data obtained from current-voltage relationship for each tested voltages, comparing oocytes expressing EAAT2 vs EAAT2 + LRRK2 and EAAT2 + LRRK2 before and after MLi-2 treatment.

Table S3

	P value
**EAAT1 vs EAAT1 + LRRK2 WT**	0.87
**EAAT1 vs EAAT1 + LRRK2 G2019S**	0.68
**DAT vs DAT + LRRK2 WT**	0.11
**DAT vs DAT + LRRK2 G2019S**	0.23
**GAT1 vs GAT1 + LRRK2 WT**	0.12
**GAT1 vs GAT1 + LRRK2 G2019S**	0.57
**BGT-1 vs BGT-1 + LRRK2 WT**	0.40
**BGT-1 vs BGT-1 + LRRK2 G2019S**	0.25
**GlyT1b vs GlyT1b + LRRK2 WT**	0.52
**GlyT1b vs GlyT1b + LRRK2 G2019S**	0.29
**GlyT2 vs GlyT2 + LRRK2 WT**	0.78
**GlyT2 vs GlyT2 + LRRK2 G2019S**	0.66

Tab S3. Statistical analysis (unpaired t-test) on raw data obtained from amplitudes of the substrate transport associated-current, comparing oocytes expressing the transporter alone and co-injected with LRRK2 WT or G2019S, at V_h=-60mV.

Table S4

I_{MAX}	P value
**EAAT1 vs EAAT1 + LRRK2**	0.89
**DAT vs DAT + LRRK2**	0.28
**GAT1 vs GAT1 + LRRK2**	0.40
**BGT-1 vs BGT-1 + LRRK2**	0.44
**GlyT1b vs GlyT1b + LRRK2**	0.84
**GlyT2 vs GlyT2 + LRRK2**	0.61

Tab S4. Statistical analysis (unpaired t-test) of I_{max} values obtained from concentration-response fitting in oocytes expressing the transporter alone and with LRRK2 exposed to increasing concentrations of the substrate, at V_h=-60mV.

Table S5

$K_{0.5}$	P value
**EAAT1 vs EAAT1 + LRRK2**	0.13
**DAT vs DAT + LRRK2**	0.58
**GAT1 vs GAT1 + LRRK2**	0.26
**BGT-1 vs BGT-1 + LRRK2**	0.73
**GlyT1b vs GlyT1b + LRRK2**	0.82
**GlyT2 vs GlyT2 + LRRK2**	0.97

Tab S5. Statistical analysis (unpaired t-test) of $K_{0.5}$ values obtained from concentration-response fitting in oocytes expressing the transporter alone and with LRRK2 exposed to increasing concentrations of the substrate, at V_h=-60mV.

References

Soreq, H., & Seidman, S. (1992). Xenopus oocyte microinjection: from gene to protein. *Methods Enzymol.*, *207:225-65*. https://doi.org/10.1016/0076-6879(92)07016-h
